# A new vessel segmentation algorithm for robust blood flow quantification from two‐dimensional phase‐contrast magnetic resonance images

**DOI:** 10.1111/cpf.12582

**Published:** 2019-06-06

**Authors:** Sebastian Bidhult, Erik Hedström, Marcus Carlsson, Johannes Töger, Katarina Steding‐Ehrenborg, Håkan Arheden, Anthony H. Aletras, Einar Heiberg

**Affiliations:** ^1^ Department of Clinical Sciences Lund Clinical Physiology Skane University Hospital Lund University Lund Sweden; ^2^ Department of Biomedical Engineering Faculty of Engineering Lund University Lund Sweden; ^3^ Department of Clinical Sciences Lund Diagnostic Radiology Skane University Hospital Lund University Lund Sweden; ^4^ Department of Health Sciences Physiotherapy Lund University Lund Sweden; ^5^ Laboratory of Computing, Medical Informatics and Biomedical – Imaging Technologies School of Medicine Aristotle University of Thessaloniki Thessaloniki Greece; ^6^ Wallenberg Center for Molecular Medicine Lund University Lund Sweden

**Keywords:** ascending aorta, interobserver variability, PC‐MRI, phantom experiments, pulmonary artery, semi‐automatic analysis

## Abstract

Blood flow measurements in the ascending aorta and pulmonary artery from phase‐contrast magnetic resonance images require accurate time‐resolved vessel segmentation over the cardiac cycle. Current semi‐automatic segmentation methods often involve time‐consuming manual correction, relying on user experience for accurate results. The purpose of this study was to develop a semi‐automatic vessel segmentation algorithm with shape constraints based on manual vessel delineations for robust segmentation of the ascending aorta and pulmonary artery, to evaluate the proposed method in healthy volunteers and patients with heart failure and congenital heart disease, to validate the method in a pulsatile flow phantom experiment, and to make the method freely available for research purposes. Algorithm shape constraints were extracted from manual reference delineations of the ascending aorta (*n* = 20) and pulmonary artery (*n* = 20) and were included in a semi‐automatic segmentation method only requiring manual delineation in one image. Bias and variability (bias ± SD) for flow volume of the proposed algorithm versus manual reference delineations were 0·0 ± 1·9 ml in the ascending aorta (*n* = 151; seven healthy volunteers; 144 heart failure patients) and −1·7 ± 2·9 ml in the pulmonary artery (*n* = 40; 25 healthy volunteers; 15 patients with atrial septal defect). Interobserver bias and variability were lower (*P* = 0·008) for the proposed semi‐automatic method (−0·1 ± 0·9 ml) compared to manual reference delineations (1·5 ± 5·1 ml). Phantom validation showed good agreement between the proposed method and timer‐and‐beaker flow volumes (0·4 ± 2·7 ml). In conclusion, the proposed semi‐automatic vessel segmentation algorithm can be used for efficient analysis of flow and shunt volumes in the aorta and pulmonary artery.

## Introduction

Phase‐contrast magnetic resonance (PC‐MR) enables non‐invasive quantification of blood flow (Nayler *et al*., 1986) and is widely used to characterize cardiovascular disease. Phase‐contrast magnetic resonance flow measurements in the ascending aorta currently serve as a reference standard for non‐invasive quantification of cardiac output (CO). Pulmonary‐to‐systemic cardiac output ratio (Qp/Qs) used to detect and quantify cardiac shunt volumes can also be accurately calculated from non‐invasive PC‐MR flow measurements in the ascending aorta and main pulmonary artery (Arheden *et al*., 1999; Petersen *et al*., 2002).

Phase‐contrast magnetic resonance blood flow measurements require delineation of the vessel of interest over the cardiac cycle. Since both the ascending aorta and pulmonary artery move within the image plane over the cardiac cycle, manual delineation in PC‐MR images is time‐consuming. This leads to a need for robust automatic or semi‐automatic segmentation algorithms. Previous vessel segmentation algorithms have effectively reduced required analysis time, and improvements have been made in regard to edge detection performance (Chwialkowski *et al*., 1996), pixelwise detection of the vessel lumen (Alperin & Lee, 2003) and the amount of required user input (Zöllner *et al*., 2009). Further, active contour tracking (Kozerke *et al*., 1999; Krug *et al*., 2003; Herment *et al*., 2010) has been applied to improve segmentation robustness. However, the need for manual correction remains.

Creating an accurate time‐resolved automatic segmentation method for PC‐MR images is a challenging task, particularly because image contrast often varies considerably over the cardiac cycle. Time phases during ventricular systole which commonly contain high blood flow velocities tend to have greater image contrast between arteries and surrounding tissue compared to time phases during ventricular diastole. Therefore, segmentation algorithms must rely on built‐in vessel shape constraints during diastolic time phases for accurate results. Previous methods have used shape constraints based on either fixed curvature or elasticity criteria within the segmentation model (Krug *et al*., 2003; Herment *et al*., 2010) or shape templates from previously completed segmentations in adjacent time phases (Kozerke *et al*., 1999).

We hypothesized that shape constraints based on a data set of manually delineated PC‐MR images can improve robustness of semi‐automatic vessel segmentation methods. Therefore, the aims of this study were to (i) develop a semi‐automatic vessel segmentation algorithm freely available for research with shape constraints based on manual vessel delineations, (ii) validate the method in phantom experiments and (iii) compare the method to an experienced observers reference delineations in human 2D PC‐MR images of the ascending aorta and main pulmonary artery.

## Methods

The Regional Ethics Review Board approved the study which also complies with the Declaration of Helsinki. The study population consisted of 231 human subjects in total (69 females; median age 53 years; age range 21–86 years). Two‐dimensional PC‐MR data of the ascending aorta from 17 healthy volunteers and 154 patients with heart failure (defined as left ventricular ejection fraction below 40%) were retrospectively included from a previous study of cardiac index (Carlsson *et al*., 2012). Two‐dimensional PC‐MR data of the pulmonary artery from 35 healthy volunteers and 25 patients with atrial septal defects were also included from a previous study of atrial septal shunt volumes (Stephensen *et al*., 2016). Vessel shape constraints and optimized algorithm parameters were extracted from 40 data sets, 20 from the ascending aorta group (10 healthy volunteers) and 20 from the pulmonary artery group (10 healthy volunteers). The remaining 191 data sets were used for algorithm evaluation.

### Image acquisition and analysis

**Figure 1:**
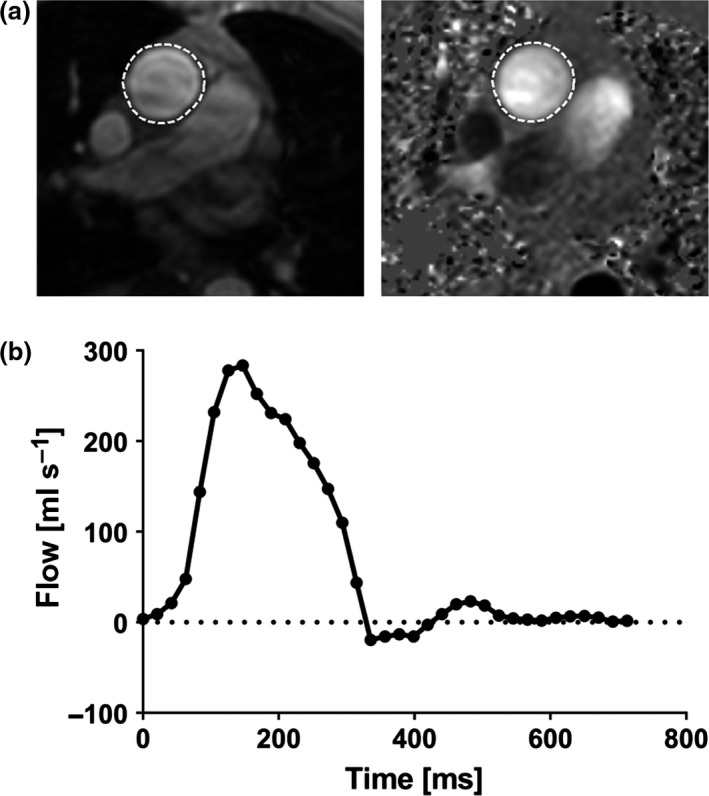
Example of a 2D PC‐MR flow volume measurement. Top panel (a) shows reference delineations (dashed white lines) of the ascending aorta in a magnitude image (left) and the corresponding phase‐contrast image (right) in early ventricular systole in a transversal image plane. The lower panel (b) shows measured flow over time after delineations in all time phases throughout the cardiac cycle. The flow volume was calculated from the flow sum over time.

The proposed segmentation algorithm was trained and evaluated for use in two‐dimensional PC‐MR human data from three 1·5T MR scanner models: Aera (Siemens, Erlangen, Germany), Magnetom Vision (Siemens, Erlangen, Germany) and Achieva (Philips, Best, the Netherlands). Validation of measured flow volumes was performed in a pulsatile flow phantom (Töger *et al*., 2015) using two scanners: one 1·5T scanner (Aera, Siemens) and one 3T scanner (Prisma, Siemens). Phase‐contrast magnetic resonance flow images of the ascending aorta were collected in a transversal slice orientation (Fig. 1a) and images of the pulmonary artery were collected in a double‐oblique orientation, both according to clinical routine. Typical sequence parameters for 2D PC‐MR pulse sequences are shown in Table S1. A temporal resolution between 16 and 30 ms and velocity encoding 200 cm s^−1^ were typically used for PC‐MR measurements. Flow data were collected using both prospectively gated sequences during free breathing (*n* = 17 subjects) and retrospectively gated sequences during both free breathing (*n* = 195 subjects) and breath‐hold (*n* = 19 subjects). Different background phase correction techniques were applied for each vendor: (i) offline linear background correction in the software Segment (Heiberg *et al*., 2010; Medviso AB, Lund, Sweden) was applied for data from Siemens scanners, and (ii) on‐scanner automatic local phase correction was automatically applied during image reconstruction on the Philips scanner. Quantification of net blood flow volume was performed by delineating the vessel of interest in all cardiac time phases and calculating the blood flow sum over time. An example flow curve over time from the ascending aorta is shown in Fig. 1b. An experienced observer performed manual reference vessel delineations in all data sets using Segment.

Algorithm development, numerical optimization and algorithm evaluation were performed in MATLAB 2014a (Mathworks Inc, Natick, MA, USA) on a personal computer (CPU clock: 2·7 GHz; RAM: 16 GB 2133 MHz DDR4).

### Extracting vessel shape profiles

Shape profiles were extracted from the training set (*n* = 40 subjects) by parameterization of shape changes over the cardiac cycle from manual vessel delineations of the ascending aorta (*n* = 20 subjects) and pulmonary artery (*n* = 20 subjects). In short, principal component analysis (PCA; Pearson, 1901) was applied to compress reference delineation data such that typical shape profiles were found (implementation details are shown in Appendix A).

### Algorithm overview

The main motivation behind the proposed method for semi‐automatic vessel segmentation was to balance robustness and flexibility so that erroneous expansion of segmentations into adjacent anatomy and image artefacts was avoided while a high degree of segmentation accuracy was obtained. The proposed method was based on a modified active contour scheme (Kass *et al*., 1988; Heiberg *et al*., 2005) which was further constrained by the new vessel shape constraints described above.

A flow chart of the method is shown in Figure S1. The algorithm was initialized by selecting a vessel of interest from a manual delineation in one time frame of the PC‐MR image series. The manual delineation was then automatically modified in the following steps:

1. Rigid motion tracking over the cardiac cycle
2. Active contour deformations using information from PC‐MR magnitude images
3. Shape‐constrained reconstruction using the compressed manual delineation data from the training set
4. Rescaling by an optimized, fixed scaling factor

Rigid motion tracking over the cardiac cycle (step 1) was performed by cross‐correlating magnitude images from adjacent cardiac time phases. The processing order of time phases for active contour deformations and shape‐constrained reconstruction (steps 2–3) was determined from the spatial median velocity inside the motion‐tracked delineation in each PC‐MR image in the following manner: the cardiac time series was divided into two classes by the *K*‐means clustering algorithm (MacQueen, 1967), one class with time phases containing high velocities and one class with time phases containing low velocities.

The time phases with high velocities were set as potential candidates for initializing active contour deformations and shape‐constrained reconstructions (steps 2–3). The manually delineated time phase was selected as the segmentation starting point if this image was included in the high‐velocity time interval. If this was not the case, the time phase corresponding to maximum median velocity was selected as starting point.

The algorithm started to process the time interval with high velocity, continued with time phases after the high‐velocity interval and completed the segmentation by processing time phases before the high‐velocity interval. Processing of a time phase/image consisted of edge guided active contour deformations derived from the PC‐MR magnitude image (step 2), followed by shape‐constrained reconstruction (step 3).

All images except the manually delineated time frame were initialized as the segmentation result from its previously processed neighbour, which was shifted according to the rigid motion tracking result.

In order to reduce systematic errors from the edge detector, the vessel segmentation diameter was rescaled with an optimized, fixed scaling factor (step 4). The rescaled segmentations were presented as the final segmentation result. Further, algorithm implementation details are shown in Appendix B.

### Parameter optimization

Numerical optimization of the segmentation method was performed to find a set of algorithm parameter values resulting in high segmentation performance. Segmentation performance was evaluated by calculating the Dice overlap coefficient (Dice, 1945) between the proposed method and reference delineations from an experienced observer, serving as the reference standard. A large Dice overlap average without an overexpressed variability was considered an indicator of high segmentation performance. All numerical optimizations were performed in the training set (*n* = 40), and the evaluated parameter combinations are summarized in Table S2. In order to evaluate the advantage of the new shape constraints, the proposed segmentation method was compared to methods utilizing shape constraints which have been used successfully in two studies for accurate aorta segmentation in MR images (Krug *et al*., 2003; Herment *et al*., 2010). The previously presented shape constraints applied a curvature force to constrain the shape of active contours, and the method was separately optimized in this study according to Table S2.

### Phantom measurements

In order to validate PC‐MR flow measurements with a largely user‐independent reference standard, flow volume measurements were performed in a custom flow phantom (Töger *et al*., 2015) consisting of a pulsatile pump connected to plastic tubing inside a water tank. Two‐dimensional PC‐MR flow volume measurements were compared to timer‐and‐beaker flow volumes obtained by measuring the total water volume output from the water tank during 2–4 min (depending on pump setting). Phantom experiments were performed at varying pump stroke volumes at 1·5T and 3T. The MR scanners were connected to the phantom pump trigger signal in order to enable gating of the MR acquisition. Two‐dimensional PC‐MR images were acquired in a transversal imaging plane through a plastic tube with 26 mm inner diameter inside the water tank. Velocity encoding was set perpendicular to the imaging plane. Regions of interest were drawn manually and by using the proposed semi‐automatic segmentation method. Sequence parameters for the phantom validation are included in Table S1.

### Statistical analysis

For human data, the proposed semi‐automatic segmentation method was compared to manual reference delineations in the test set (*n* = 191) containing time‐resolved delineations of the ascending aorta in 151 human subjects and delineations of the pulmonary artery in 40 human subjects. For this comparison, the proposed segmentation algorithm was initialized by one manually delineated time phase corresponding to 20% of the RR interval. The Dice overlap coefficient was used to measure segmentation overlap with reference delineations, and blood flow volumes were compared using modified Bland–Altman analysis (Bland & Altman, 1986) with manual reference delineations serving as reference standard. Bias and variability between two methods were defined as mean and one standard deviation (1 SD), respectively. The 95% limits of agreement (LoA) were defined as mean ± 1·96 SD, and the Wilcoxon signed‐rank test was used to test statistical significance of differences between paired observations. A significance level of *P*≤0·05 was considered statistically significant. The performance evaluation was repeated for two versions of the algorithm, one version with the new shape constraints (the proposed method) and one version with previously presented shape constraints (a curvature force for active contours), as described in more detail above. The performance impact of initializing the proposed segmentation method at different parts of the cardiac cycle was evaluated by repeating the comparison between the proposed method and reference delineations for algorithm initialization in 20 equidistant time phases over the RR interval. Interobserver variability was determined using Bland–Altman analysis (Bland & Altman, 1986) in a randomly selected subset of the test set including 15 aorta and 15 pulmonary artery data sets. For manual reference delineations, two experienced observers manually outlined all images, and for the proposed semi‐automatic method, the same two observers outlined the vessel in one image. Quantification of cardiac shunts by measurement of the Qp/Qs ratio (pulmonary‐to‐systemic cardiac output ratio) was performed in a subset of the test set (*n* = 25 subjects) using (i) the proposed semi‐automatic method initialized at 20% of the RR interval and (ii) manual reference delineations. Cardiac index (CI) was calculated as the aorta flow volume multiplied by heart rate and divided by body surface area (BSA).

## Results

### Parameter optimization

Optimal parameter values from numerical optimization in the training set (*n* = 40 human subjects) are shown in Table S2 (right column). During numerical optimization, Dice coefficient variability (standard deviation) increased when the proposed segmentation algorithm was tuned to reproduce complex vessel shapes. Therefore, an algorithm configuration with strict vessel shape constraints was used as optimal solution for the proposed method.

### Flow volume measurements in humans: bias and variability of the proposed method

**Figure 2:**
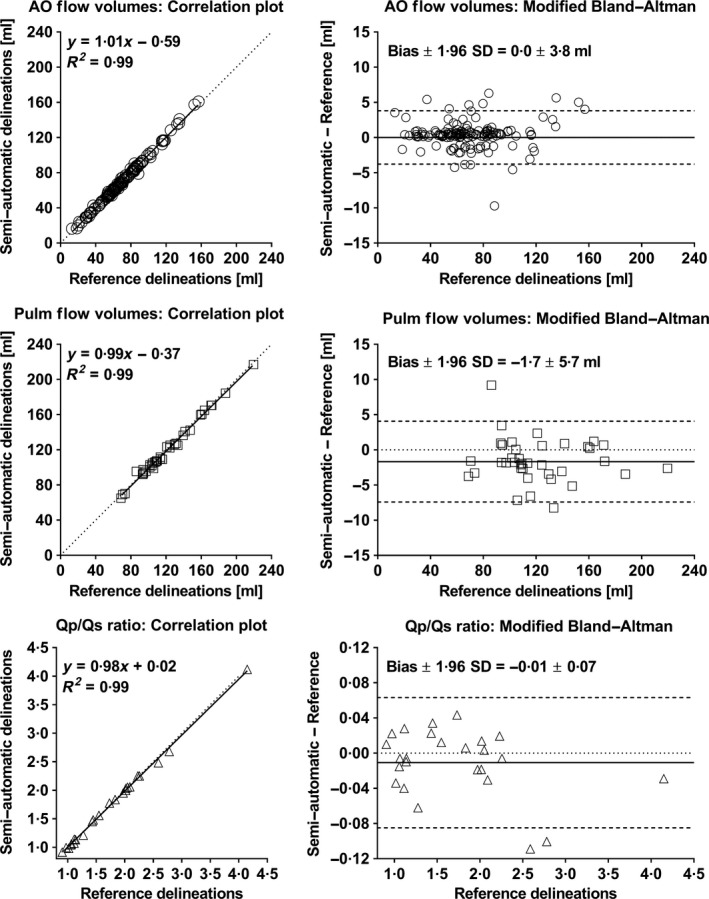
The semi‐automatic segmentation method agreed with reference delineations for flow volumes in the aorta (top panel; *n* = 151 subjects), flow volumes in the pulmonary artery (middle panel; *n* = 40 subjects) and Qp/Qs ratio calculations (bottom panel; *n* = 25 subjects). Left panels show correlation plots between semi‐automatic and manual measurements. Dotted lines indicate lines of identity, and solid lines indicate linear regressions. Right panels show modified Bland–Altman analysis for semi‐automatic and manual measurements. Dotted lines indicate zero difference between compared methods, solid lines indicate bias, and dashed lines indicate 95% limits of agreement (LoA). Low bias and variability were found for the proposed segmentation method compared to reference delineations for flow volume measurements in both aorta and pulmonary artery and for Qp/Qs ratio. AO, ascending aorta; Pulm, pulmonary artery.

Semi‐automatic flow measurements in the ascending aorta using the proposed segmentation method with algorithm initialization at 20% of the RR interval resulted in low bias and variability for flow volume (mean ± 1SD) of 0·0 ± 1·9 ml or 0·7 ± 3·7% (Figure 2; top right panel). Corresponding Dice coefficient median was 95·3% with range 68·4–97·6%. Bias and variability for cardiac index were 0·0 ± 0·06 l min^−1^ m^−2^. The processing time of the proposed segmentation method ranged from 1·7 to 6·7 s.

Results for semi‐automatic measurements in the pulmonary artery with algorithm initialization at 20% of the RR interval are shown in Figure 2 (middle panel). Low bias and variability for flow volume were obtained (−1·7 ± 2·9 ml or −1·2 ± 3·0%), and Dice coefficient median was 93·9% with range 87·8–97·3%. The computation time for the pulmonary artery ranged between 1·3 and 3·1 s.

Measurements of the Qp/Qs ratio (Figure 2; bottom panel) from semi‐automatic and reference delineations were in close agreement (*n* = 25 subjects), with bias and variability −0·01 ± 0·04 (Figure 2; bottom right panel).

### Flow volume measurements in humans: comparison between the proposed method and a previously presented semi‐automatic segmentation method

**Figure 3:**
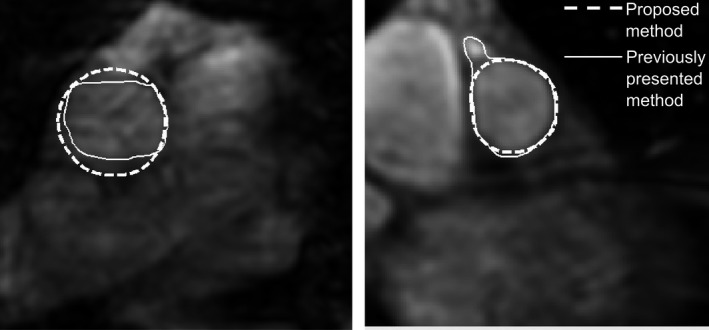
Improvement in segmentation accuracy using the proposed shape constraints in two example cases. The left image shows a transversal image slice used for flow measurement in the ascending aorta, and the right image shows a double‐oblique image slice used for flow measurements in the pulmonary artery, both in ventricular diastole. Semi‐automatic inaccurate segmentations using an optimized active contour curvature force for shape constraints are shown as solid white lines. Improved semi‐automatic segmentations using the proposed method with shape‐constrained reconstruction are shown as dashed white lines.

Semi‐automatic vessel segmentation with the new shape constraints (the proposed method) resulted in reduced absolute flow volume error compared to semi‐automatic segmentation using previously presented shape constraints (1·5 ± 1·7 ml versus 2 ± 5·4 ml; *P* = 0·007). The effect of the new shape constraints on segmentation quality is demonstrated in Figure 3.

### Flow volume measurements in humans: stability of the proposed method with respect to initialization time point

**Figure 4:**
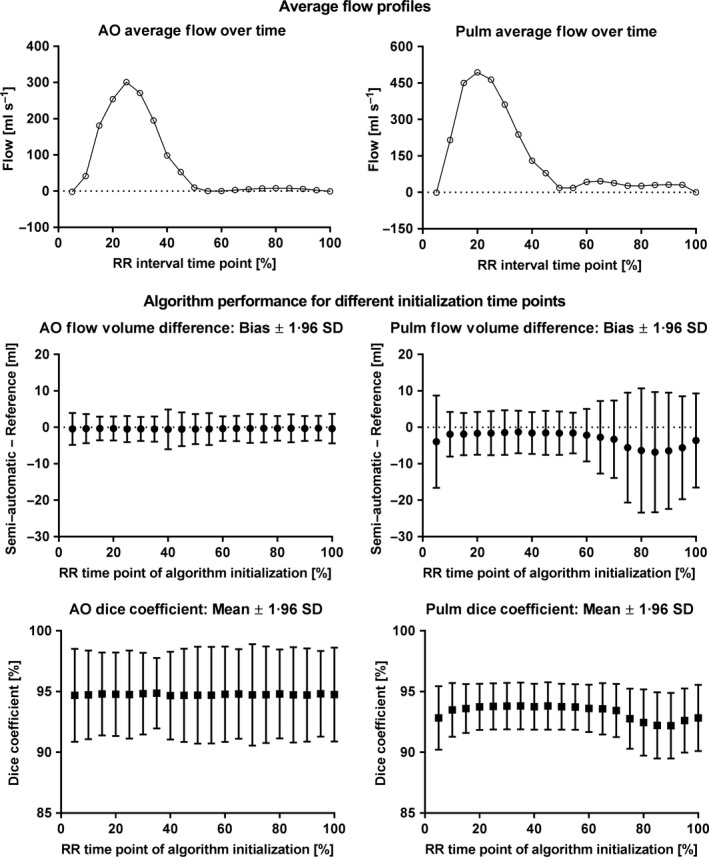
The proposed segmentation method resulted in similar performance when initialized at different time points of the RR interval. Top panel shows flow profiles over the RR interval averaged over all subjects for the ascending aorta (left) and the pulmonary artery (right). Middle panel shows flow volume bias and 95% limits of agreement (LoA; filled circles and error bars) of the semi‐automatic method versus reference delineations. Bottom panel shows average Dice coefficients and 95% limits of agreement (LoA; filled squares and error bars). The proposed segmentation method resulted in similar flow volume bias (filled circles; middle left panel), flow limits of agreement (error bars; middle left panel) and Dice coefficient performance (bottom left panel) when initialized at different time points for the ascending aorta. For the pulmonary artery, however, flow volume bias and limits of agreement were slightly sensitive to the time point of initialization (middle right panel). Pulmonary artery segmentations initialized within 10–55% of the RR interval showed stable flow volume bias (filled circles; middle right panel), flow volume limits of agreement (error bars; middle right panels) and Dice coefficient performance (bottom right panel). AO, ascending aorta; Pulm, pulmonary artery.

Figure 4 shows changes in flow volume bias and variability, and average Dice coefficients and Dice variability when the proposed semi‐automatic method was initialized at different parts of the cardiac cycle for the ascending aorta and the pulmonary artery. For the ascending aorta, the proposed segmentation algorithm was robust to different initialization time points with worst‐case flow volume bias and variability of −0·6 and 2·8 ml, obtained with algorithm initialization at 40% of the RR interval. The 95% limits of agreement for flow volume differences were within ±6 ml for algorithm initialization over the entire RR interval.

For the pulmonary artery, segmentation performance was slightly sensitive to the selected RR interval initialization time point (Figure 4), resulting in a worst‐case flow volume bias of −6·8 ml for initialization at 85% of the RR interval and worst‐case variability of 8·7 ml for initialization at 80% of the RR interval. However, initializing segmentations at 10–55% of the RR interval resulted in worst‐case flow volume bias and variability limited to −1·9 and 3·1 ml. In the majority of cases, 10–55% of the RR interval corresponded to high image contrast between the pulmonary artery and its surroundings. The 95% limits of agreement for flow volume differences were within ±8 ml for algorithm initialization within 10–55% of the RR interval.

### Flow volume measurements in humans: interobserver variability of the proposed method

**Figure 5:**
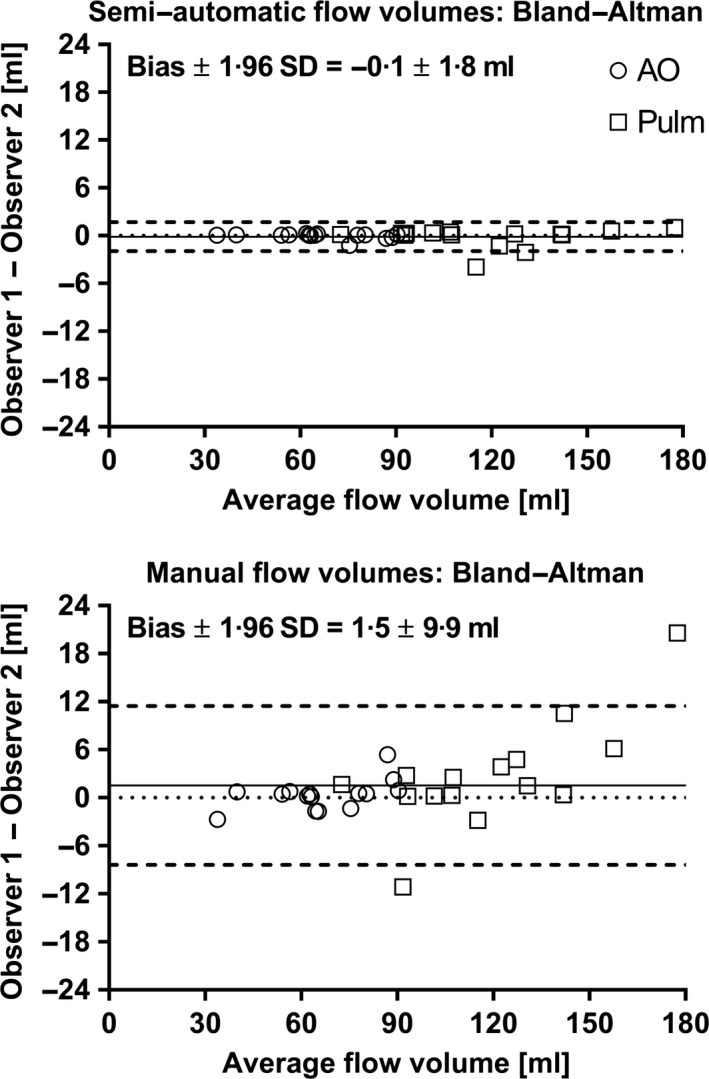
Interobserver variability of flow volumes for the proposed semi‐automatic method (top panel) and manual delineations between two observers (bottom panel). Both panels show Bland–Altman analysis. Dotted lines indicate zero flow volume difference, solid lines indicate bias, and dashed lines indicate 95% limits of agreement (LoA). A clear reduction in interobserver variability of measured flow volumes was observed for the proposed semi‐automatic method compared to manual delineation. AO, ascending aorta (open circles); Pulm, pulmonary artery (open squares). The required time of analysis for an experienced observer was approximately 2 min for manual delineation and approximately 10 s for semi‐automatic delineation.

Figure 5 shows interobserver variability from 30 human subjects for the proposed method and manual delineations. Interobserver variability was significantly lower (*P* = 0·008) for the proposed semi‐automatic method (−0·1 ± 0·9 ml) compared to manual delineations (1·5 ± 5·1 ml).

### Phantom measurements

**Figure 6:**
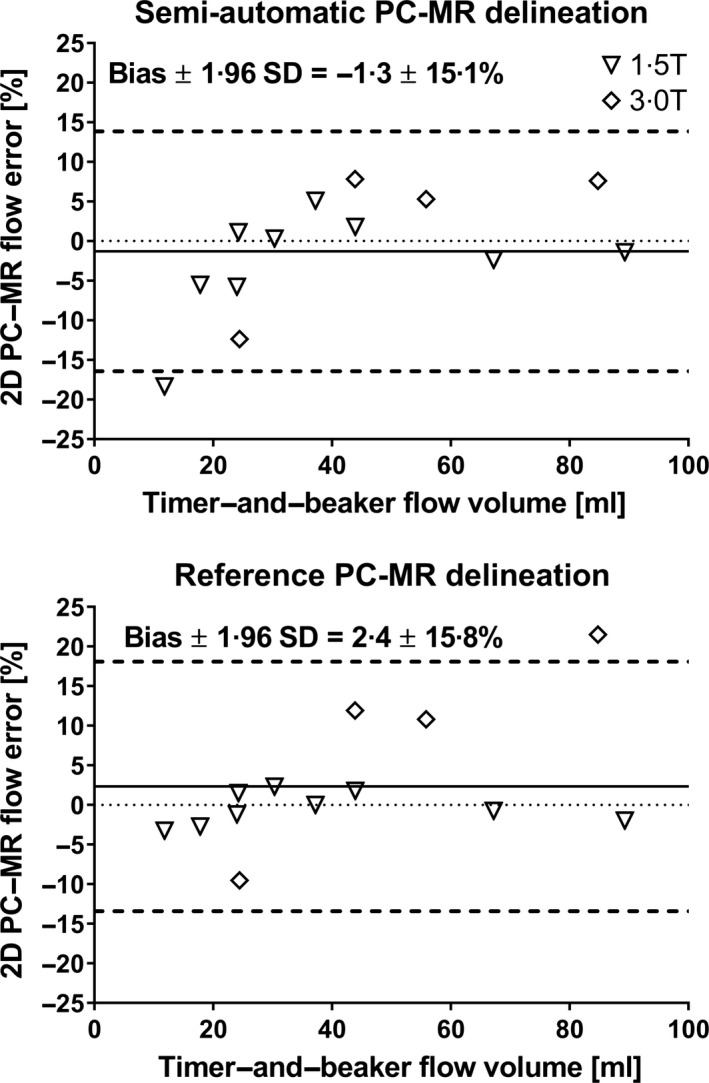
Validation of PC‐MR flow measurements in a pulsatile phantom experiment. The plots show modified Bland–Altman analyses comparing timer‐and‐beaker flow measurements with flow from 2D PC‐MR using the proposed semi‐automatic delineation method (top panel) and by manual delineation (bottom panel). For both delineation methods, PC‐MR was in close agreement with timer‐and‐beaker measurements at 1·5T (open triangles) and 3T (open squares).

Phantom timer‐and‐beaker measurements resulted in flow volumes ranging between 11·8–89·3 ml (1·5T) and 24·4–84·8 ml (3T). Agreement between 2D flow volume measurements and timer‐and‐beaker was found for both manual delineation (2·0 ± 5·5 ml or 2·4 ± 8·1%) and the proposed semi‐automatic segmentation method (0·4 ± 2·7 ml or −1·3 ± 7·7%) for both field strength (Figure 6).

## Discussion

This study presents an algorithm for semi‐automatic segmentation of the ascending aorta and the pulmonary artery in 2D PC‐MR images with new shape constraints based on reference delineation training data. The algorithm is freely available for research. The new algorithm resulted in improved vessel segmentation performance compared to a previously presented segmentation method. Good agreement was found between the proposed method and reference delineations in the ascending aorta and the pulmonary artery for both flow volume measurements and shunt volume quantification (Qp/Qs ratio) in a large population of patients and healthy subjects, demonstrating clinical applicability of the proposed method. Bias and variability for cardiac index and Qp/Qs ratio were 0·0 ± 0·06 l min^−1^ m^−2^ and −0·01 ± 0·04, respectively. These values suggest that the proposed segmentation method can be used clinically, as bias and variability are low compared with differences in cardiac index between healthy subjects and patients with congestive heart failure (3·2 ± 0·5 l min^−1^ m^−2^ versus 2·3 ± 0·6 l min^−1^ m^−2^; Carlsson *et al*., 2012) and compared with differences in Qp/Qs ratio at rest between healthy subjects and patients with an atrial septal defect (1·02 ± 0·02 versus 2·04 ± 0·17; Stephensen *et al*., 2016).

Automated vessel segmentation methods for PC‐MR flow have the potential of improving efficiency in the clinical setting by reducing analysis time and inter‐ and intra‐observer variability. Previous semi‐automatic algorithms have shown clear improvements in processing speed (Chwialkowski *et al*., 1996), low interobserver variability compared to reference delineations (Herment *et al*., 2010) and high accuracy in phantom validation (Kozerke *et al*., 1999).

### Performance of the proposed method

Segmentation of the ascending aorta was robust independent of RR time point of initialization while segmentation of the pulmonary artery required an initialization time point at 10–55% of the RR interval for accurate results. The variation in vessel diameter of the pulmonary artery during the cardiac cycle is generally larger compared to the ascending aorta. This may in part explain the observed differences between the two vessels. The variation in pulmonary artery vessel diameter is related to the relatively short distance between the right ventricular outflow tract and the pulmonary trunk bifurcation combined with the curvature of the pulmonary artery, making the positioning of pulmonary artery flow scan planes more challenging. Further, considering the size and shape variation of the pulmonary artery over the cardiac cycle, increased acquired temporal resolution may improve segmentation accuracy regardless of time point of algorithm initialization. However, as the optimal initialization time points (10–55% of RR) for pulmonary artery segmentation were associated with high image contrast between the pulmonary artery and its surroundings, the first step of manually delineating the pulmonary artery in the proposed semi‐automatic method should be advocated to be performed in this time interval.

The proposed method resulted in good agreement with timer‐and‐beaker flow volume measurements in a pulsatile flow phantom experiment at two field strengths and a wide range of flow volumes.

### Comparison with earlier studies and future work

The obtained limits of agreement for flow measurements in both the aorta and pulmonary artery were small in relation to reported flow volume errors caused by potential background velocity offset errors (Gatehouse *et al*., 2010).

Manual user input is required in one image for initialization of the proposed segmentation method. A previous study has demonstrated a method for automatic identification of the ascending and descending aorta in 2D PC‐MR images, assuming a strictly circular vessel lumen (Goel *et al*., 2014), which is not applicable on non‐circular structures such as the pulmonary artery. Bratt *et al*. (2019) recently provided proof of concept for a promising deep learning algorithm for fully automatic time‐resolved segmentation of aortic blood flow with similar bias and variability as the current study. Future studies may investigate potential advantages of deep learning‐based segmentation algorithms for automatic segmentation of PC‐MR images in routine clinical work.

Further, future work may include extending the proposed segmentation algorithm for use with 4D flow PC‐MR data. An atlas‐based segmentation algorithm for 4D flow PC‐MR which used *a priori* data has shown good performance in the aorta and pulmonary artery (Bustamante *et al*., 2015). Shape constraints similar to the ones used in the proposed method may further benefit automatic segmentation algorithms for 4D flow data.

### Limitations of the proposed method

In cases with complex vessel shapes not included in the training data, or with substantial image artefacts due to respiratory motion, the proposed algorithm may not be able to correctly segment the vessel of interest. Figure S2 shows two examples of such cases in the pulmonary artery and ascending aorta. Improved ability to reproduce complex vessel shapes was tested by tuning the shape constraints of the algorithm to preserve more shape variation. However, such a tuning resulted in reduced segmentation robustness during numerical optimization of the algorithm and was therefore avoided in favour of stricter shape constraint settings.

Impact of the composition of selected training data on segmentation performance was not explored in this study. Selection of training data may alter segmentation performance and may limit adequate segmentation for pathology types not included in the training set, particularly when a training set of modest size is used. The current training set is, however, representative of a clinical cohort, and the method is shown to be robust by the low number of outliers.

## Conclusion

A new semi‐automatic segmentation algorithm for MRI flow measurements in the ascending aorta and the pulmonary artery was developed and validated. Good agreement was found with reference delineations by experienced observers, and interobserver variability was reduced compared to reference delineations. In summary, the results demonstrate that the algorithm can be used for efficient and robust flow and shunt volume quantification in the clinical setting.

## Conflict of interest

Einar Heiberg is the major shareholder of Medviso AB, which produces cardiovascular imaging software. No funding for this study has been received from any non‐public funding source. No other author has any competing interests to disclose.

## Author's contributions

SB contributed to the design of the study, developed and implemented the algorithms, analysed and interpreted results, and performed reference delineation of the pulmonary artery data for interobserver variability and drafted the manuscript. EHed contributed to the design of the study and to the automatic algorithm and performed reference delineations of the aorta and pulmonary data. MC contributed to the design of the study and performed reference delineations of the ascending aorta data from normal volunteers and patients with heart failure. JT acquired the phantom data and contributed to the design of the algorithm. KSE contributed to the design of the study and collected the normal subject's data. HA contributed to the design of the study and assisted in conceiving the study. AHA contributed to the design of the study and to the design of the automatic algorithm and provided in‐depth cardiac MRI knowledge needed for algorithm development. EHeib contributed to the design of the automatic algorithm and conceived the study. All authors revised the manuscript for important intellectual content and have read and approved the final version of the article.

## Supporting information


**Figure S1.** Flow chart of the proposed semi‐automatic segmentation method. The method is initialized by a reference delineation in one time point and continues with rigid motion tracking and interleaved active contour deformations using magnitude images and shape‐constrained reconstruction. The algorithm ends by enlarging the segmentation diameter using a numerically optimized, fixed scaling factor.

**Figure S2.** Image artefact examples resulting in degraded segmentation quality for the pulmonary artery (Pulm) and ascending aorta (AO). Top panel shows magnitude images from each data set with manual (red) and semi‐automatic (blue) segmentations at a time point that demonstrates segmentation errors. Bottom panel shows corresponding phase‐contrast images at the same time point. The example for the pulmonary artery (left) demonstrates underestimation of the vessel lumen during ventricular diastole due to an imaging plane which is not strictly orthogonal to the vessel cross section. The example for the ascending aorta (right) demonstrates diverging segmentation during ventricular diastole due to respiratory motion artefacts.

**Table S1.** Typical 2D PC‐MR sequence parameters for *in vivo* and phantom data acquisitions.

**Table S2.** Optimized algorithm parameters for the proposed segmentation method (rows 1–5). A near‐identical segmentation method using previously presented shape constraints (rows 6–10) was included for comparison to evaluate the added benefits of using the new shape constraints of the proposed method. Optimal vessel diameter scaling factors (rows 5 and 10) were individually determined for all combinations of the three other algorithm parameters (rows 2–4 and rows 7–9).

## Appendix A

### Details on extracting typical vessel shape profiles from the training set and how shape constraints were implemented for the proposed segmentation method

The proposed segmentation method utilizes shape constraints to improve robustness of the method. The shape constraints are constructed from a training data set of manually delineated time‐resolved vessel segmentations in 2D PC‐MR images. In this section, lower‐case letters represent scalar numbers, bold lower‐case letters represent vectors, and bold upper‐case letters represent matrices.

In order to achieve coordinate correspondence, vessel shape changes over the cardiac cycle were parameterized by transforming the reference delineation coordinates into radial distances, sampled at 80 equidistant angles and normalized by the mean radial distance over time. The time resolution was resampled to 40 linearly spaced time phases over a cardiac cycle to ensure equidistant sampling of a cardiac cycle for all data sets. Shape profiles were stored in a matrix **R** with size 40 × 3200 (i.e. including 40 subjects and 80 delineation nodes in 40 time phases), and decomposition of the data into linearly uncorrelated components was performed using principal component analysis (PCA; Pearson, 1901) of **R**. This decomposition enabled lossless reconstruction of a resampled vessel segmentation data set from the weighted sum of all principal components, according to Eqs 1 and 2:

(1) $$r=m+\sum_{i\;=\;1}^{n}a_{i}v_{i};$$

(2) $$a_{i}=v_{i}\cdot {\left(r-m\right)}^{T}=\sum_{j\;=\;1}^{n}r(j)v_{i}\left(j\right);$$

Here, ***m*** denotes the data set average‐shape column vector, *a*_*i*_ indicates the weight for the *i*th principal component ***v***_***i,***_ ***r*** is the fully reconstructed data set (column vector) and *n* is the number of principal components. If a shape profile is reconstructed from only a subset of principal components, the result will approximate the original data set and will be constrained by shape profiles contained within the selected principal components. Finding the weights for a subset of principal components from Eq. 2 can be rewritten as a solution to a linear least‐squares regression problem:

(3) $$\hat{r}=r-m;$$

(4) $$V_{k}=\left[v_{1},\;v_{2},\;\ldots .,\;v_{k}\right];$$

(5) $$\hat{a}=\overset{\mathrm{argmin}}{a}{||\hat{r}-V_{k}a\left|\right|}_{2}^{2}=\left(V_{k}^{T}V_{k}\right)\;{}^{-1}V_{k}^{T}\hat{r}={\mathrm{IV}}_{k}^{T}\hat{r}=V_{k}^{T}\hat{r}$$

Here, upper‐case letters represent matrices and lower‐case letters represent column vectors. ***V***_*k*_ denotes the *k* selected principal components (column vectors) with 1−*k*:th largest variance and ***I*** is the identity matrix. This method may be used to apply shape constraints on time‐resolved vessel segmentations. The number of principal components in use (*k*) was determined from numerical optimization. An estimation of the segmentation mask centre of mass was needed to convert segmentation node coordinates into radial distances before imposing shape constraints. In order to approximate the centre of mass of a segmentation result containing erroneous outlier nodes, all segmentation masks were eroded using a binary two‐dimensional disc with a 4‐pixel radius before calculating the centre of mass.

## Appendix B

### Algorithm implementation details

In this section, lower‐case letters represent scalar numbers, bold lower‐case letters represent vectors, and bold upper‐case letters represent matrices.

#### Rigid motion tracking in magnitude images

The user‐defined delineation was tracked through the image time series by a two‐dimensional local cross‐correlation algorithm. An analysis window surrounding the reference delineation was extracted in magnitude image data from two adjacent time phases. The data were then resampled using bilinear interpolation with a factor of 2 and cross‐correlation performed by conjugate multiplication in the Fourier domain. The spatial shift resulting in maximum correlation was chosen as the detected vessel translation between adjacent time phases.

#### Active contour deformation model

The implemented active contour model is based on the original formulation by Kass *et al*. (1988) and was implemented in part in two previous studies (Heiberg *et al*., 2005; Tufvesson *et al*., 2015). The active contour used the following energy minimization problem for a given segmentation ***v***(*s*):

(6) $$\overset{arg\;min}{v}e=\int_{0}^{1}e\left(v\left(s\right)\right)\text{d}s=\int_{0}^{1}e_{\mathrm{int}}\left(v\left(s\right)\right)+e_{\mathrm{ext}}\left(v\left(s\right)\right)\text{d}s$$

Here, ***v***(*s*) is represented by a set of equidistantly spaced discrete node points along a closed contour. The internal energy term, *e*_int_, imposes shape constraints on the segmentation while the external energy term, *e*_ext_, enables attraction and repulsion from various image features. A local energy minimum is found by solving the Euler–Lagrange differential equation:

(7) $$0=\frac{\text{d}}{\text{d}s}\left(\frac{\text{d}}{\text{d}v_{s}}e_{\mathrm{int}}\right)+\frac{\text{d}}{\text{d}v_{s}}e_{\mathrm{ext}}=Ku+f\left(u\right)$$

The two derivative terms represent internal and external forces that control the segmentation evolution over multiple iterations. The equation is converted into a finite difference scheme in the right‐hand side of equation (7), where ***u*** represents the node point coordinates, the stiffness matrix **K** corresponds to internal forces, and *f*(***u***) represents forces associated with external image features at node point coordinates ***u***. The external force was based on edge detection in PC‐MR magnitude images. The internal force of the proposed method was set to zero and replaced by the a‐priori shape‐constrained reconstruction, described in Appendix A, which was performed once after active contour deformations.

#### Edge detection in magnitude images

The edge detection was implemented as four separate one‐dimensional edge detectors with filter kernel [−1 2 −1] at different orientations rotated 45° from each other, creating one horizontal, one vertical and two diagonal filter kernels. Convolution with the filter kernels was preceded by horizontal and vertical smoothing of the magnitude images using the filter kernel [0·25 0·5 0·25]. For each node point, the active contour model used a weighted combination of the two filter directions resulting in the largest and second‐largest scalar product with the local delineation normal vector, as previously proposed (Heiberg *et al*., 2005). Using this method, nodes on the closed curve were attracted to strong image gradients along their local normal vectors.
