# Supplementary figures and images for: A new vessel segmentation algorithm for robust blood flow quantification from two‐dimensional phase‐contrast magnetic resonance images
Source: Clin Physiol Funct Imaging. 2019 Jun 6;39(5):327–38. doi: 10.1111/cpf.12582 (PMC6852024; doi:10.1111/cpf.12582)

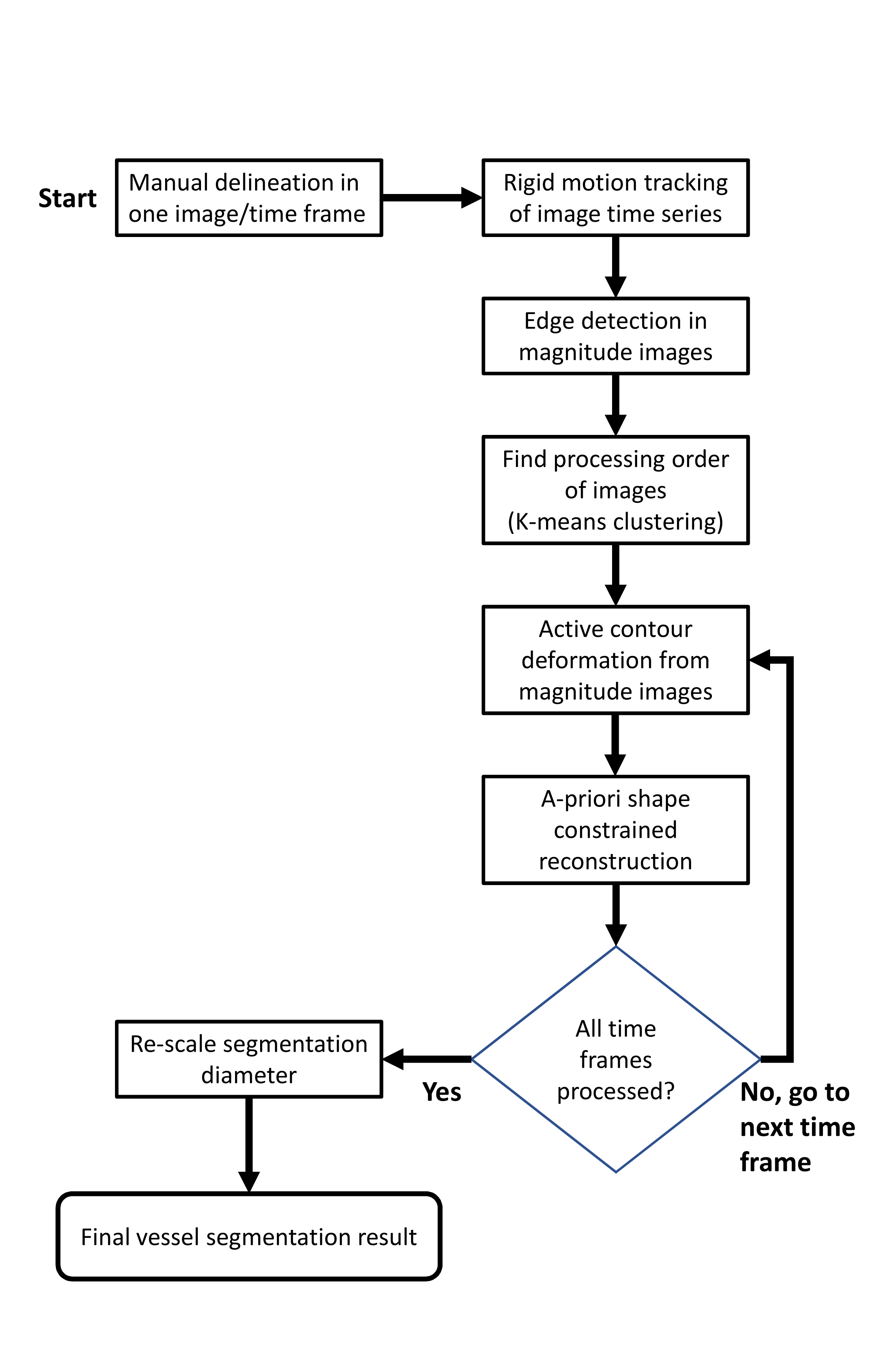

Supplement: Supplementary file 1 — Figure S1. Flow chart of the proposed semi‐automatic segmentation method. The method is initialized by a reference delineation in one time point and continues with rigid motion tracking and interleaved active contour deformations using magnitude images and shape‐constrained reconstruction. The algorithm ends by enlarging the segmentation diameter using a numerically optimized, fixed scaling factor. [file CPF-39-327-s001.PNG]

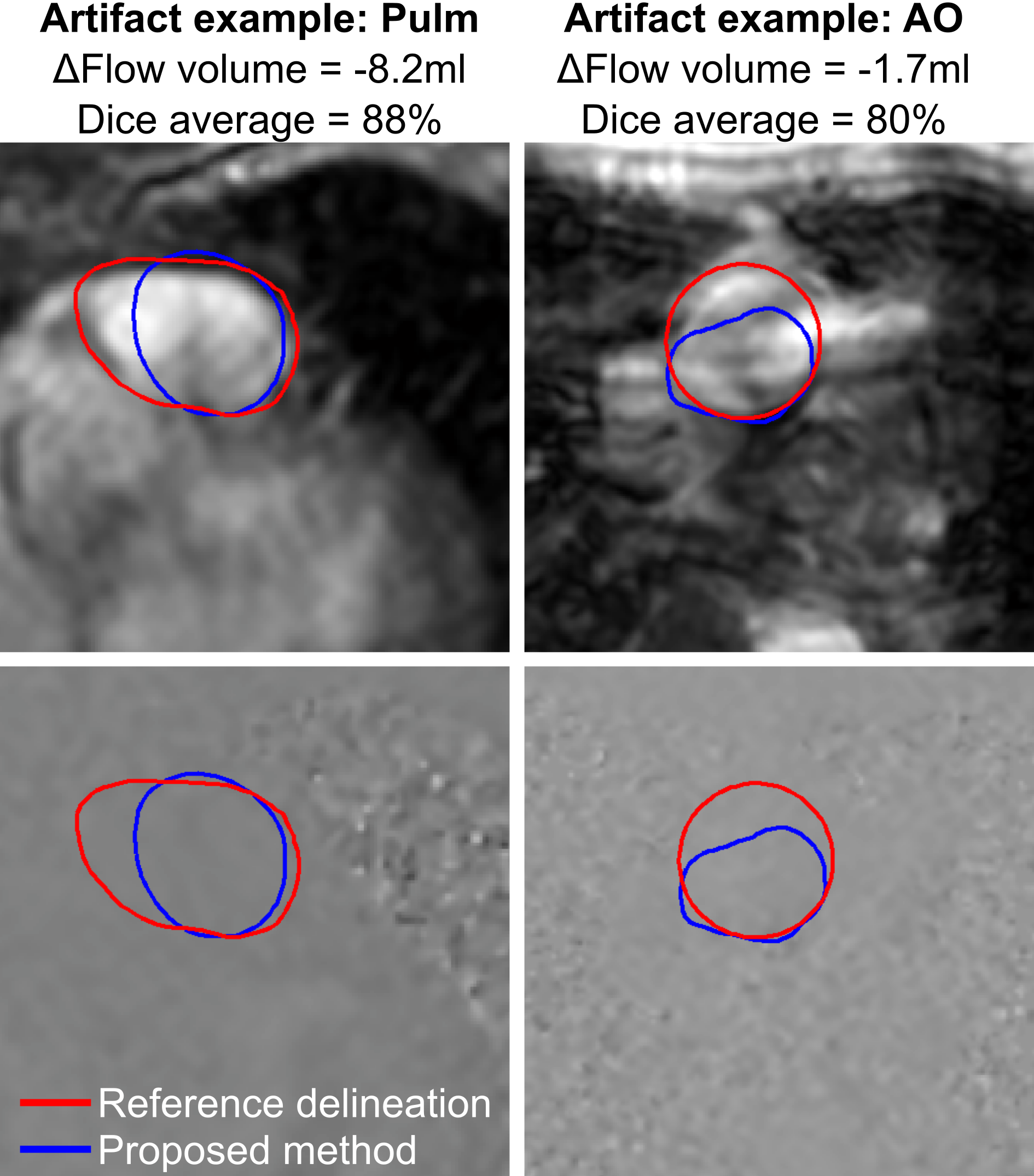

Supplement: Supplementary file 2 — Figure S2. Image artefact examples resulting in degraded segmentation quality for the pulmonary artery (Pulm) and ascending aorta (AO). Top panel shows magnitude images from each data set with manual (red) and semi‐automatic (blue) segmentations at a time point that demonstrates segmentation errors. Bottom panel shows corresponding phase‐contrast images at the same time point. The example for the pulmonary artery (left) demonstrates underestimation of the vessel lumen during ventricular diastole due to an imaging plane which is not strictly orthogonal to the vessel cross section. The example for the ascending aorta (right) demonstrates diverging segmentation during ventricular diastole due to respiratory motion artefacts. [file CPF-39-327-s002.png]
