# Supplementary material for: A new vessel segmentation algorithm for robust blood flow quantification from two‐dimensional phase‐contrast magnetic resonance images
Source: Clin Physiol Funct Imaging. 2019 Jun 6;39(5):327–38. doi: 10.1111/cpf.12582 (PMC6852024; doi:10.1111/cpf.12582)
Supplement: Supplementary file 3 — Table S1. Typical 2D PC‐MR sequence parameters for in vivo and phantom data acquisitions. [file CPF-39-327-s003.docx]

**Web Supplemental Table 1**. Typical 2D PC-MR sequence parameters for in-vivo and phantom data acquisitions

| MRI sequence parameters | In-vivo data (Philips 1.5T) Free-breathing non-segmented retrospective gating  (n= 191 subjects) | In-vivo data (Philips 1.5T) Breath-hold segmented retrospective gating  (n= 19 subjects) | In-vivo data (Siemens 1.5T) Free-breathing non-segmented prospective gating  (n= 17 subjects) | In-vivo and phantom  (Siemens 1.5T) Free-breathing  non-segmented retrospective  gating  (n= 4 subjects) | Phantom data (Siemens 3T)  non-segmented retrospective  gating  (phantom only) |
| --- | --- | --- | --- | --- | --- |
| Echo time [ms] | 5.3 | 2.3 | 5 | 2.7 | 3.0 |
| Slice thickness [mm] | 6 | 10 | 8 | 5 | 5 |
| Time resolution [ms] | 17.3 | 15.7 | 30 | 19.6 | 21.4 |
| VENC [cm/s] | 200 | 200 | 250 | 200 | 200 |
| Flip Angle [°] | 15 | 15 | 30 | 20 | 20 |
