# Supplementary material for: A new vessel segmentation algorithm for robust blood flow quantification from two‐dimensional phase‐contrast magnetic resonance images
Source: Clin Physiol Funct Imaging. 2019 Jun 6;39(5):327–38. doi: 10.1111/cpf.12582 (PMC6852024; doi:10.1111/cpf.12582)
Supplement: Supplementary file 4 — Table S2. Optimized algorithm parameters for the proposed segmentation method (rows 1–5). A near‐identical segmentation method using previously presented shape constraints (rows 6–10) was included for comparison to evaluate the added benefits of using the new shape constraints of the proposed method. Optimal vessel diameter scaling factors (rows 5 and 10) were individually determined for all combinations of the three other algorithm parameters (rows 2–4 and rows 7–9). [file CPF-39-327-s004.docx]

**Web Supplemental Table 2**. **Optimized algorithm parameters for the proposed segmentation method (rows 1-5).** A near-identical segmentation method using previously presented shape constraints (rows 6-10) was included for comparison to evaluate the added benefits of using the new shape constraints of the proposed method. Optimal vessel diameter scaling factors (rows 5 and 10) were individually determined for all combinations of the three other algorithm parameters (rows 2-4 and rows 7-9).

| Optimized algorithm parameters:  **Proposed segmentation method with new shape constraints** | Evaluated parameter values: [min, max] Δ = interval spacing | Determined  optimal value |
| --- | --- | --- |
| Number of iterations | [5, 35]; Δ = 10; | 35 |
| Edge detection parameter | [0.5, 4]; Δ = 0.5; | 1.5 |
| Preservation of shape variance [%] | [50, 100];  Δ = 10 (from 50 to 90); Δ = 1 (from 92 to 100); | 50% |
| Diameter scaling factor | Not applicable | 1.06 |
| Optimized algorithm parameters:  **Segmentation method with previously presented shape constraints** | Evaluated parameter values: [min, max] Δ = interval spacing | Determined  optimal value |
| Number of iterations | [5, 35]; Δ = 10; | 15 |
| Edge detection parameter | [0.5, 4]; Δ = 0.5; | 3 |
| Curvature parameter  (Not used in the proposed method) | [0.2, 0.5] Δ = 0.1 | 0.3 |
| Diameter scaling factor | Not applicable | 1.08 |
